# Inflammation drives tumor growth in an immunocompetent implantable metastasis model

**DOI:** 10.21203/rs.3.rs-4719290/v1

**Published:** 2024-08-11

**Authors:** Connor Giles, Jungwoo Lee

**Affiliations:** University of Massachusetts Amherst; University of Massachusetts Amherst

## Abstract

Nearly 90% of cancer deaths are due to metastasis. Conventional cancer therapeutics including chemotherapy, surgery, and radiotherapy, are effective in treating primary tumors, but may aggravate disseminated tumor cells (DTCs) into regaining a proliferative state. Models isolating the post dissemination environment are needed to address the potential risks of these therapies, however modeling post dissemination environments is challenging. Often, host organisms become moribund due to primary tumor mass before native metastatic niches can evolve. Implantable tissue engineered niches have been used to attract circulating tumor cells independent of the primary tumor. Here, we serially transplant such tissue engineered niches with recruited DTCs in order to isolate the post dissemination environment. After transplantaion, 69% of scaffolds developed overt post-dissemination cancer growth, however 100% of scaffolds did not grow to a life-threatening critical size within twelve weeks. Adjuvant chemotherapy, while initially effective, did not prevent long-term DTC growth in scaffolds. Subjecting these transplanted niches to surgical resection via biopsy punch enhanced CD31, MMP9, Ly6G, and tumor burden compared to control scaffolds. Biopsy punching was able to rescue tumor incidence from prior chemotherapy. This model of serial transplantation of engineered DTC niches is a highly controllable and flexible method of establishing and systematically investigating the post-dissemination niche.

## Introduction

In 2022, it was estimated that there were just under 300,000 new breast cancer cases^1^. Conventional therapies are effective in reducing or eliminating primary tumors, however, metastases can develop years or decades later in tissues far from the primary tumor. Such metastatic cancer is the cause of 90% of cancer deaths^2–4^. Prior to metastatic tumor growth, disseminated tumor cells (DTCs) must first successfully engraft in these distant metastatic sites and then, eventually, regain a rapidly proliferating phenotype. However, relatively little is known about how dormant DTC’s regain their proliferative phenotype. There is a staggering lack of models that can capture the dormant niche and the critical transition in DTC’s to a proliferative state, limiting progress towards effective treatment for disseminated cancer^5^.

Cancer-environment interaction is pivotal in understanding metastatic progression. The seed and soil hypothesis, first described by Paget in 1889, posits that successful metastasis depends on cancer cell “seeds” modifying the “soil” of the distant microenvironment. The bottleneck of microenvironment modification as described by the seed and soil hypothesis means, clinically, that circulating tumor cells (CTC’s) largely invade a select few tissues such as the bone, brain, liver, and lung in the case of breast cancer. The specificity with which CTC’s invade secondary sites points to a robust intercommunication between cancer cells and these distant niches. Before colonizing distant tissue, cancer cells modify these secondary metastatic sites extensively through secretion of growth factors and extracellular vesicles which enhance vascularization, inflammation, ECM remodeling, and inflammatory cell recruitment^6^,^7^. Through these modifications, tumors form pre-metastatic niches (PMN’s) in secondary sites that are amenable to tumor cell colonization in which CTC’s invade. Rather than immediately begin proliferating, the cancer cells become DTC’s and remain dormant for years.

Evidence suggests that the primary breast tumor begins shedding tumor cells early on in its development, which may limit future therapeutic strategies focused on preventing metastasis^8–10^. Furthermore, there is a significant body of evidence that conventional cancer treatments enhance dissemination^11^,^12^. Instead, developing therapeutic strategies centered around preventing DTC’s from regaining a proliferative phenotype or simply eliminating DTC’s before then may prove to be more feasible. Still, this strategy faces a multitude of challenges. DTC’s are often highly resistant to therapy^13–16^. A number of studies have shown it is possible to overcome this resistance and sensitize these dormant cancer cells to further treatment in certain circumstances^17^. Alternatively, rather than target DTC’s themself, it may be more effective to reeducate their microenvironment to eliminate pro-tumorigenic features such as inflammation and tumor derived ECM composition^18^. Regardless, developing more effective strategies depends on accurate modeling of the clinical scenario in which DTC’s inhabit a realistic secondary environment.

Navigating these therapeutic hurdles necessitates new models that accurately mimic the clinical scenario. A surgical intervention model demonstrated that mice with implanted polyvinyl acetate sponges to mimic wounds had a tumor incidence of 60% as compared to unwounded mice with 10% suggesting that surgery-driven inflammation is implicated in tumor growth ^19^. Administration of anti-inflammatory meloxicam resulted in tumor diameter dropping from 5mm down to 2mm, again suggesting inflammation as the critical mediator in tumor incidence. Mechanistically, proinflammatory molecules such as S100A9 have been linked to tumor cell reactivation via myeloid derived suppressor cell activity^20^. In-situ management of the tumor environment has similarly revealed that local anti-inflammatory administration and immunomodulatory drugs can decrease local recurrence following tumor resection^21^,^22^. Anti-recurrence treatment on the basis of niche regulation is a potentially valuable therapeutic strategy, however a model capable of translating such results to scenarios of distant metastasis is needed to develop such strategies.

Contrastingly, tissue engineered PMN’s leverage material-host interaction to simulate distant tissue colonization.^23–25^. Tissue engineered models are capable of recruiting immune cells, cancer cells, and stromal cells to develop realistic PMN’s with highly tunable properties including ECM composition and pore size^26–31^. For example, researchers have taken ECM from metastatic tissues and coated engineered scaffolds to provide a more realistic environment while maintaining the scaffold’s ability to support DTC’s^26^. In this work, the researchers decellularized lung tissue with sodium dodecyl sulfate, minced, lyophilized, dissolved in a solution of pepsin and 0.1 M of hydrochloric acid, and coated onto PCL scaffolds. Such artificial PMN’s enable deep investigation into the processes by which CTC’s infiltrate the niche, become dormant, and re-emerge later, replicating the critical junctures in metastatic cancer progression^32^.

While it has been demonstrated that implanted PMN’s are capable of rapid CTC’s accumulation^33^, long-term PMN evolution is comparatively less understood. Long-term models are required in order to fully investigate emerging evidence that conventional therapies may actually hasten the rate at which DTCs regain proliferative capabilities^12,34–41^. Negative niche changes due to treatment such as inflammation and ECM remodeling are implicated in driving metastasis^42,43^. In surgical wounding models, targeting inflammatory pathways or otherwise using anti-inflammatory drugs and immune landscape engineering abrogated the increase in tumor burden following surgery^19,21–23,44^. Often, long-term PMN study is limited by host animal morbidity before metastatic recurrence can be observed. A compelling solution to this critical limitation is serially transplanting the niche to a secondary host to isolate the post-dissemination niche from the primary tumor. This serial transplantation strategy allowed for the observation of the effect that subsequently injected peripheral blood mononuclear cells had on the post-dissemination niche^45^. Models specifically designed to enable investigation of such disruptions within the post dissemination cancer niche are needed to understand how treatments can potentially hasten metastatic recurrence.

In this paper, we aim to develop and demonstrate a model of disseminated cancer using serially transplanted hydrogel materials to sequester circulating tumor cells. It is demonstrated that these transplantable cancer niches are capable of recruiting cancer cells, which retain proliferative capacity after serial transplantation. In order to investigate the effects of inflammation on the post dissemination niche, we biopsy punched serially transplanted scaffolds. We conducted this biopsy punch procedure, additionally, on scaffolds that had been exposed to doxorubicin in order to more closely mimic post-treatment clinical scenarios. These studies were conducted using immunocompetent mice in order to maintain the immunological dimension of the DTC niche which many models must eliminate. Together, this data suggests that this model system obviates key limitations in studying the post dissemination niche, and enables deep investigation into the dynamics of DTC’s.

## Methods and Materials

### Polyacrylamide inverted crystal colloid hydrogel scaffold fabrication

Soda lime glass beads were sorted using an Advantech Sonic Sifter. Beads were dispersed in deionized (DI) water. Five layers of glass beads size 250μm-300μm were placed in glass tubes sized 8 × 35mm. The glass tubes were sonicated in an ultrasonic waterbath in order to arrange the glass beads into a regular crystalline pattern. The packed beads were dried at 60°C in an oven. The beads were then annealed together in a furnace at 664°C for 4 hours. A solution of 30wt% polyacrylamide (fisher catalog BP170) and 1.5wt% bis-acrylamide crosslinker were dissolved in deionized water. 0.2vol% 2-hydroxy-2-methylpropiophenone photoinitiator and 0.2 vol % N,N,N′,N′-tetramethylethylenediamine accelerator were added to the solution. The solution was purged with nitrogen gas for 30 minutes to eliminate oxygen. 150μL of polyacrylamide precursor solution was infiltrated into the templates via centrifugation at 9,000

RPM for 15 minutes. Infiltrated templates were polymerized under a 15W UV lightsource for 20 minutes. Polymerized templates were removed from the exterior glass tube the following day to ensure complete polymerization. Excess hydrogel was removed with a razorblade such that the annealed glass beads were exposed. The exposed glass beads were dissolved via solution of hydrofluoric acid diluted in 1.2M hydrochloric acid at a ratio of 1:5. Hydrofluoric acid washes were alternated with washes of 2.4M hydrochloric acid. Each wash was conducted on a shake plate over 4 hours. The shake plate was placed within a fume hood, and samples were handled with proper protective gear to minimize the risk from these corrosive washes. After dissolution, the scaffolds were washed three times with DI water to remove any residual acid. The washed scaffolds were lyophilized. Scaffolds were sterilized with 70% ethanol for 1 hour. Sterile scaffolds were surface treated with sulfosuccinimidyl 6-(4’-azido-2’-nitrophenylamino)hexanoate (Sulfo-SANPAH). Sulfo-SANPAH surface-treated scaffolds were immersed in 10% rat tail collagen solution. The scaffolds and collagen solution were poured into a petri dish in a biosafety cabinet and exposed to UV light for 30 minutes. After 30 minutes, the scaffolds were flipped and exposed to UV light for an additional 30 minutes. After UV-crosslinking the collagen to the Sulfo-SANPAH, the scaffolds were immersed in collagen solution overnight. Scaffolds were stored in sterile PBS at 4°C.

### Subcutaneous scaffold implantation into a MMTV-PyMT+ mouse

A breeding pair of MMTV-PyMT mice (Jackson Labs 002374) were obtained from Jackson Laboratories. Mice were housed in sterile conditions with unrestricted access to food and water. Mice used in this study were between 4 and 13 weeks of age. PyMT mice were anesthetized with 1.5% isoflurane. Electric clippers and nair were used to remove the dorsal hair. The skin was sterilized with 70% isopropyl alcohol wipes. Mice received 2 mg meloxicam/kg mouse weight subcutaneously. A subcutaneous pocket was created by making a small horizontal incision approximately 2 mm in length and expanding surgical scissors in the dorsal space. One scaffold was placed in the pocket, and the incisions were closed using two 7mm wound clips that were removed one week later. Each mouse received 4 such implants. Each mouse received 4 such implants. In compliance with IACUC guidelines, animals were euthanized prior to tumor size reaching a critical threshold of 1,000 mm^3^.

### Serial transplantation of implanted scaffolds to a secondary MMTV-PyMT^−^ mouse

For serial transplantations from one mouse to another, the initial mouse was euthanized and the implants were immediately transferred to PBS prior to reimplantation in a secondary mouse. Transplanted materials were placed within subcutaneous pockets as with the initial implantation.

### Surgical instigation of early tumor microenvironments by in-situ biopsy punch

Biopsy punch experiments were carried out 2 weeks after serial transplantation. A small horizontal incision proximal to the subcutaneous pocket was created. Scissors were used to sever any tissue connecting the scaffold to the muscle layer. Sterile, blunt forceps were used to grasp the scaffold and invert the subcutaneous pocket. A sterile 3mm biopsy was used to remove the center of the hydrogel scaffold. Hydrogel scaffold of the same dimensions replaced the removed scaffold center. The subcutaneous pocket was uninverted and the incision was closed using 7mm wound clips which were removed one week later.

### Chemotherapy simulation

In primary mice, doxorubicin was injected intraperitoneally at approximately 12 weeks of age. Dosage was 2.5mg/kg. Stock solution of 10mg/mL was diluted in sterile PBS (10%) down to a concentration of 0.5mg/mL. In order to achieve a dosage of 5mg/kg, 10μL per gram mouse was injected e.g. for a 35g mouse, 350μL were injected.

### Histological tissue analysis

Tissue samples were cryopreserved by embedding in Cryomatrix embedding resin and snap freezing in a container with 2-methylbutane and dry ice. Frozen tissue was sectioned using a Cryostat (NX70). Scaffolds were cut to 30μm thick sections. Cryopreserved samples were stored at −80°C.

For hematoxylin and eosin staining, frozen tissue sections were fixed with 10% neutral buffered formalin for 10 minutes prior to staining. The formalin was washed with DI, and slides were stained with dye solution according to manufacturer protocols (American Master Tech).

### Immunofluorescent staining

For immunohistochemistry, slides were fixed in pre-chilled acetone for 10 minutes. The slides were then washed three times in wash buffer composed of PBS with 0.05% Tween-20. Fixed samples were outlined with hydrophobic marker and blocked with 10% goat serum and 1% BSA for 2 hours at room temperature. Primary antibodies were diluted in blocking solution at a ratio of 1:200 and left overnight at 4°C. Samples were then washed an additional 3 times with wash buffer followed by the addition of secondary antibodies diluted in blocking solution at a ratio of 1:200 and left for 2 hours at room temperature. Samples were washed 3 times in wash buffer and 1μg/mL DAPI solution was added. Stained samples were imaged immediately. Rat-anti mouse PyMT (Abcam ab15085) was used for primary antibody labeling of cancer cells. Goat-anti rat 488 (Life technology A-11006) was used as the secondary antibody. DAPI (vector labs H1200) was used as a nuclear counterstain.

### Statistical Analysis

Unpaired student’s t tests were performed to compare the means of two experimental groups. Data were considered statistically significant if p < 0.05 for two tailed analysis. Quantitative data represent mean and standard error.

## Results

### Implantable pre-metastatic niche (PMN) attracts circulating tumor cells released from a breast tumor developed in MMTV-PyMT + female mice.

**Figure 1:**
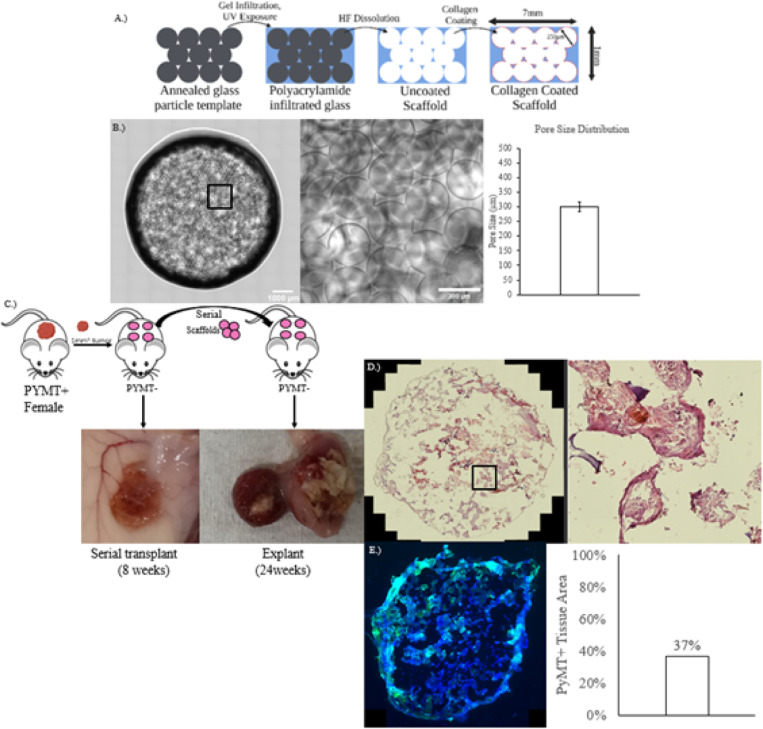
Inverse Crystal Colloid hydrogel scaffold fabrication and implantation. A. Fabrication process of scaffolds. B. Hydrogel scaffold surface (left), closeup (center), and pore size distribution (right). C. Serial transplantation schematic (above), representative scaffold prior to serial transplantation (below, left), scaffold explanted after 24 weeks (below, right). D. H&E section, and closeup of tissue-infiltrated pore (right).E. IHC stain of PyMT, counterstained by DAPI.

This initial experiment was carried out in order to ascertain whether or not this hydrogel based PMN model would be able to shelter DTC’s until they form tumors in secondary hosts after transplantation. The scaffold was engineered such that it would recruit circulating tumor cells, a necessary function of a PMN (Fig. 1A). The scaffold accomplishes this by leveraging its geometrical and chemical properties to instigate an intense foreign body response. Firstly, the spherical, regular pores, enhance surface area dramatically compared to bulk material (Fig. 1B). The high surface area affords the maximum region in which the scaffold can interact with surrounding tissue. Secondly, the synthetic polyacrylamide hydrogel is a non-degradable material, enabling the foreign body response to persist. The robust foreign body response instigated by the implanted material drives vascularization and tissue infiltration (Fig. 1C).

Four ICC polyacrylamide scaffolds were fabricated as previously described and implanted subcutaneously in female mouse at 4 weeks of age (Fig. 1C). Two weeks after the scaffolds, a tumor 1mm^3^ tumor piece was orthotopically implanted. After this tumor grew to a critical size of 1000mm^3^, the scaffolds were serially transplanted to a secondary mouse. The mouse was euthanized and the scaffolds were explanted when a tumor growth was observed to have reached the critical size of 1000mm^3^, which took 24 weeks following transplantation. IHC was used to visualize PyMT + tumor cells within the scaffold environment. Immunohistochemical staining was performed on the scaffold, and 37% of the tissue area was PyMT+.

### Serial transplantation of implantable PMNs to MMTV-PyMT^−^ female mice allows long-term evolution of metastatic tumor microenvironment.

In order to accurately model recurrence, it is necessary for the scaffold to facilitate DTC quiescence over extended periods of time while still allowing for subsequent outgrowth of palpable tumors. The evolution of the disseminated niche and the switch from dormant tumor cell to actively proliferating metastatic lesion is poorly understood, and thus a highly valuable process to emulate. Therefore, serial transplantation was used to obviate host morbidity and investigate the kinetics of the DTC niche.

**Figure 2:**
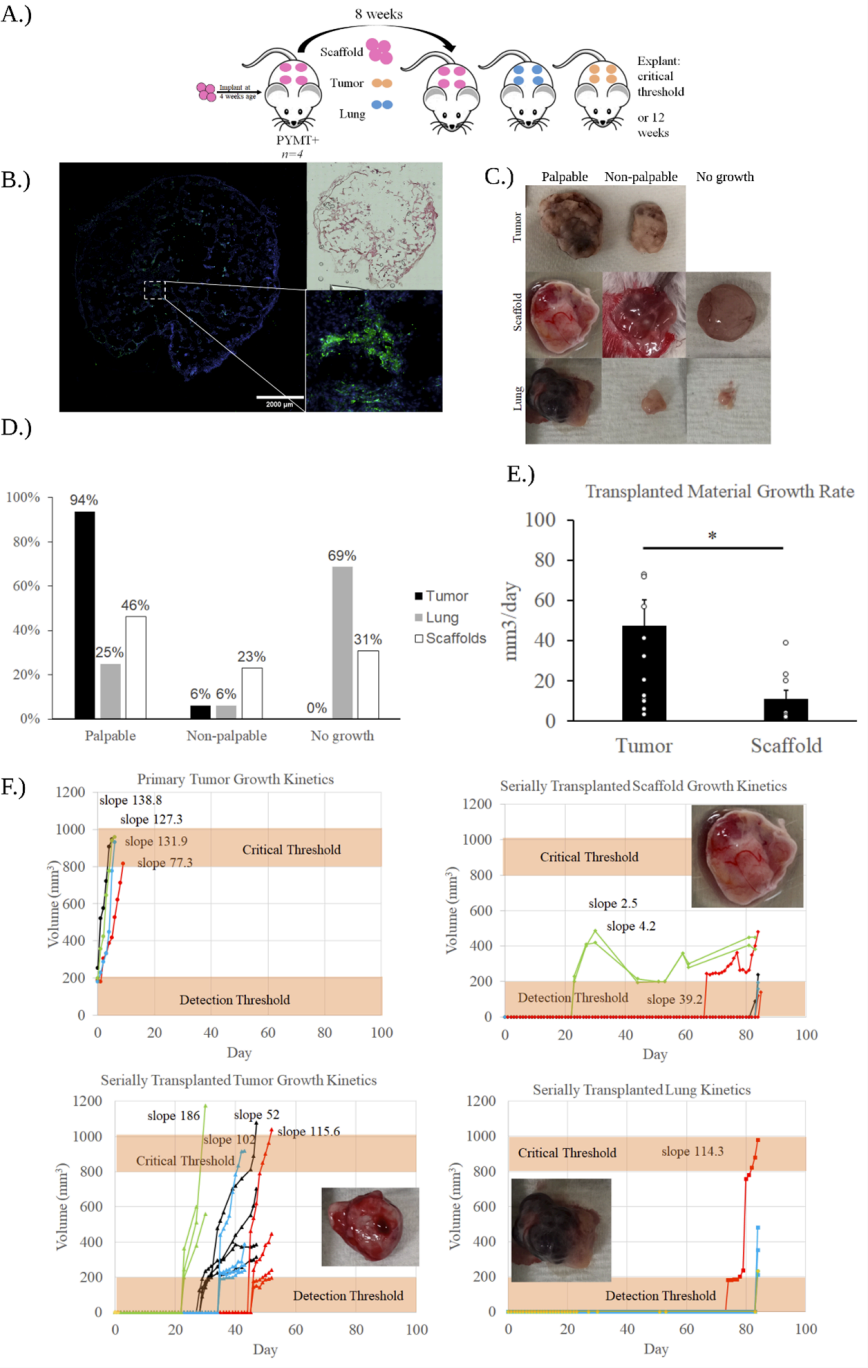
Growth behavior and kinetic analysis of serially transplanted materials. A. timeline of transplantation study. B. Immunohistochemical stain confirming DTC presence. C. Representative images of serially transplanted material classifications.D. Distribution of growth profiles of serially transplanted materials. E. growth rate comparison between serially transplanted tumors and scaffolds. F. Growth kinetics of primary tumors and serially transplanted materials.

Primary MMTV-PyMT mice were implanted with scaffolds at 4 weeks of age (Fig. 2A). These mice developed spontaneous tumors at 10–12 weeks of age. Scaffolds were serially transplanted when the host mouse’s primary tumor reached a critical threshold of 1000mm^3^. To compare engraftment and growth kinetics of the scaffolds to typical metastatic tissue, lung from the host mouse was also implanted serially, and pieces of the primary tumor were also implanted as control.

Immunohistochemistry was used to confirm the presence of DTC’s in serially transplanted materials (Fig. 2B). Serially transplanted materials were classified into one of three growth profiles (Fig. 2C). Transplanted materials are classified as palpable tumor growths when their growth can be observed without invasive means such as incision. Palpable growths are able to be measured via calipers whereas the other growth profiles maintain their original dimensions subcutaneously. Palpable tumor growth was certain beyond a detection threshold of approximately 200mm^3^. Non-palpable growths were those in which there was visually apparent evidence of tumor growth on the scaffold surface, but did not expand the subcutaneous pocket, and thus was not measurably different from the starting size. Non-palpable tumor growths could only be identified after an incision or explantation. The final category was no growth. Such transplanted materials had no visually identifiable trace of cancer growth. Transplanted materials were classified into these groups and compared (Fig. 2D). Over the course of the study period, the volume of materials was recorded. Serially transplanted tumors grew at an average rate of 51.5mm^3^/day compared to serially transplanted scaffolds which grew at an average rate of 13mm^3^/day (Fig. 2E). Volumes of serially transplanted materials over the study period were recorded over time (Fig. 2F). Primary tumors reached the critical threshold of 1000mm^3^ in a number of days. All serially transplanted tumors had one implant which reached the critical. Growth was continuous from onset which began at a range from approximately 20 days to upwards of 40 days post transplantation. The majority of lung transplants either did not grow past the critical threshold or were completely degraded. One transplanted lung reached the critical threshold which began growing at nearly 70 days. No serially transplanted scaffolds reached the critical threshold within 12 weeks. Three scaffolds grew above the detection threshold starting from approximately 20 days to over 60 days.

## Discussion

The inverse crystal colloid distribution of the hydrogel scaffolds generates a large surface area upon which the scaffold can interact with host tissue. The high surface area results in a strong foreign body response, which is critical for recruiting blood vessels, CTC’s and other relevant PMN elements. The initial implantation study (Fig. 1C) validated the scaffold was capable of recapitulating the full metastatic cycle from CTC recruitment to critical tumor outgrowth. This full lifecycle would not be possible without serial transplantation; as demonstrated by the primary tumor growth kinetics (Fig. 2F), primary tumors lead to host death rapidly. Serial transplantation allows for the separation of the PMN from the primary tumor.

Kinetic data indicates that a scaffold environment is necessary for long-term niche observation. In scaffolds, onset of cancer growth began as early as 20 days and as late as 60 days. However, these palpable cancer growths did not reach the critical threshold within the 12 week study period, and thus can be considered dormant for purposes of this study. In all palpable tumor type scaffold growths, there was a *decrease* in size at some point within the study period, suggesting potential immune-mediated dormancy, where the immune system keeps tumor proliferation in check^46^, though the mechanism driving this observed decrease is currently unclear. In primary tumor pieces, the critical threshold was reached every time soon after an apparent escape from dormancy. While there was an initial lagtime of 20–40 days that may yield insight into DTC behavior, it is also possible that this delay was an effect of transplantation, and no real dialogue occurred between the transplanted cancer cells and their niche. Furthermore primary cancer cells are distinct from cancer cells that are capable of metastasis^47^,^48^. Thus, the lung tissue is a more realistic comparison given that it is a typical site of metastasis for breast cancer^49^. However, the growth profile distribution (Fig. 2D) demonstrates the limitations with the approach. Host tissue failed to develop significant cancer growth following transplantation in the majority of cases. While tumor tissue was identifiable in all samples, the majority did not grow past the detection threshold. The kinetic data indicates that host tissue alone is insufficient to provide a supportive niche for studying DTC outgrowth. There have been strategies to obviate this limitation. In previous work, host tissue was combined with ICC polyacrylamide hydrogel scaffold by biopsy punching the scaffold and filling the gap with host tissue^50^. Similarly to the findings presented in this paper, host tissue without the supportive scaffold had significantly fewer DTC’s. This was attributed to the lack of factors that the scaffold supplied including immunomodulatory effects such as increased recruitment of neutrophils and robust vascularization caused by FBR-driven inflammation leading to tissue collapse. In line with this previous work, our data suggests that scaffolds afford a stable tissue environment for DTC’s that allow for a significant period of dormancy.

**Figure 3:**
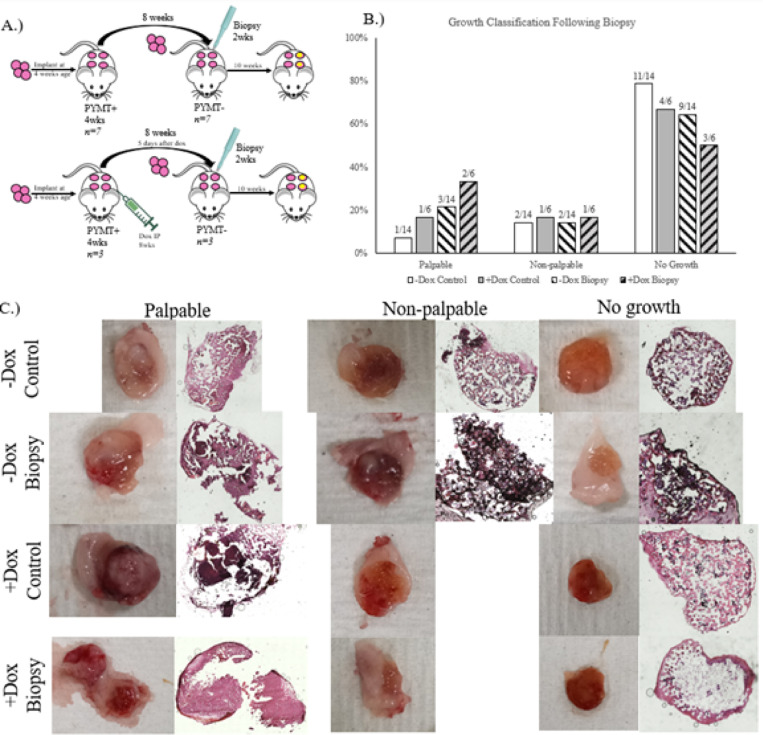
In-situ Instigation of PMN following chemotherapy. A. Serial transplantation and biopsy schematic without chemotherapy (top) and with chemotherapy (bottom). B. Classification of explanted materials. C. Representative images of each growth classification for each group.

In the clinic, chemotherapy is administered to treat the primary tumor, which can have negative effects such as selecting populations of cancer cells for enhanced survivability^12^. Tumor growth indicated that biopsy punching is capable of enhancing tumor growth following chemotherapeutic treatment (Fig. 3B). Chemotherapy exposed, biopsied scaffolds had the highest fraction of palpable, large solid tumor development of any group (Fig. 3C). This suggests that chemotherapy treatment prior to serial transplantation does not inhibit DTC survival to a degree where they can no longer develop tumor growths. It may even be possible that chemotherapy primes the DTC niche to be more receptive to the inflammation caused by the biopsy punch afterwards, however further research is needed to evaluate this claim.

In contrast to much metastasis research, these studies were conducted with immunocompetent mice. PDX and other humanized models offer translational opportunities by using human cells, however they risk losing effects caused by immune interactions. Biomaterials scaffolds are highly capable of modulating the immune environment^32^, potentially allowing for the investigation of phenomenon such as immune-mediated dormancy which may be a critical in recurrence^54^.

Unusually, lung transplantation demonstrated consistent failure to develop growths despite lung tissue being a common metastatic site. Since lung tissue was harvested from primary tumor bearing mice that were implanted with scaffolds, it is possible that the scaffolds shielded the lung from dissemination or otherwise altered the typical metastatic progression of the lung tissue. Such shielding of organs by implantable PMN’s has been previously demonstrated^27^, indicating that this may be an explanation worth further investigation.

The first and foremost limitation of this study is the lack of consensus and clarity on what constitutes dormancy. Dormancy can be characterized as long periods of no growth or limited growth depending on the scenario, or growth kept in check by limited vascularization or immunosurveillance^55^. The kinetic data of transplanted scaffolds (Fig. 2F) indicated limited, slow growth of DTC’s over the study period, and this has been taken to be indicative of dormancy for the purpose of this study, however it is debatable whether or not this is representative of clinical scenarios. Furthermore, the mechanism by which DTC growth is constrained in the scaffold environment is not fully detailed. Further analysis is needed to determine what factors such as gel stiffness, ECM composition, vascular development, or otherwise are arresting DTC growth as compared to unconstrained tumor piece transplant.

Future research may investigate the effect of ECM on niche evolution. Previous work has highlighted the effect of chemotherapy-induced ECM alteration and how increased collagen-IV and fibronectin enhance invasion^26^,^56^. Additionally, ECM components such as fibronectin enable cellular dormancy via $\alpha_{5}\beta_{1}$ integrin binding^57,58^. While the scaffolds in this study have all been coated with collagen, the same process of sulfo-SANPAH surface chemistry is capable of producing scaffolds coated with alternative ECM proteins such as fibronectin.

Observing long term DTC niche evolution involves overcoming many obstacles including host morbidity, CTC collection, and niche stimulation. We present this model as a method of overcoming such limitations and enabling investigation of critical topics including inflammation and ECM remodeling in the DTC niche.

## Conclusion

Our implantable hydrogel model attracts CTC’s which maintain a stable environment for extended periods of time. In-situ inflammation drives tumor recurrence in this model environment. The systematic investigation of the post-dissemination niche this model enables may alter the standard of care for metastatic cancer.

## Data Availability

Data were generated by the authors and available on request.
